# Efficient CO_2_ and H_2_O Co-Electrolysis in a BaZr_0.44_Ce_0.36_Y_0.2_O_3‑δ_-Based Proton Ceramic Electrochemical Reactor with BaGd_0.8_La_0.2_Co_2_O_6‑δ_ Steam Electrodes

**DOI:** 10.1021/acsaem.5c03443

**Published:** 2026-01-15

**Authors:** Elena Barrio-Querol, Imanol Quina, Maria Fabuel, Kwati Leonard, Hiroshige Matsumoto, José Manuel Serra, Laura Almar, Sonia Escolástico

**Affiliations:** † 83167 Instituto de Tecnología Química (Universitat Politècnica de València-Consejo Superior de Investigaciones Científicas), Av. Los Naranjos s/n, 46022 València, Spain; ‡ Center for Energy Systems Design (CESD), International Institute for Carbon-Neutral Energy Research (I2CNER), Kyushu University, 744 Motooka, Nishi-ku, Fukuoka 819-0395, Japan

**Keywords:** CO_2_ and H_2_O co-electrolysis, proton ceramic electrochemical reactor (PCER), BGLC steam electrodes, H_2_ production, process intensification

## Abstract

Despite the increasing use of electrification and efforts to decarbonize processes, hydrocarbons remain indispensable for heavy transport and chemistry sectors. Electrochemical devices powered by renewable energy offer a promising strategy to convert CO_2_ into sustainable molecules that can be used as fuels and chemical feedstocks. In this study, we investigate the electrochemical performance of BaGd_0.8_La_0.2_Co_2_O_6‑δ_ as a steam or oxygen electrode in a proton ceramic electrolysis cell. Two composite materials were evaluated as electrode candidates, consisting of BaGd_0.8_La_0.2_Co_2_O_6‑δ_ (BGLC) combined with either BaZr_0.4_Ce_0.4_Y_0.1_Yb_0.1_O_3‑δ_ (BZCYYb4411) or BaZr_0.5_Ce_0.3_Y_0.2_O_3‑δ_ (BZCY532). The electrodes were characterized using electrochemical impedance spectroscopy (EIS) on symmetrical cells under various oxidizing conditions and temperatures. Based on this analysis, the BGLC-BZCYYb4411 electrode was selected as the steam electrode in a full PCER configuration, composed of Ni-SrZr_0.5_Ce_0.4_Y_0.1_O_2.95_ (SZCY541) as the fuel/CO_2_ electrode and BaZr_0.44_Ce_0.36_Y_0.2_O_3‑δ_ as the electrolyte. The cell was evaluated for co-electrolysis of H_2_O and CO_2_. The results illustrate the potential of the BGLC-based electrodes and BZCY-based electrolytes for efficient CO_2_ upgrading and steam electrolysis in a single-step process.

## Graphical abstract

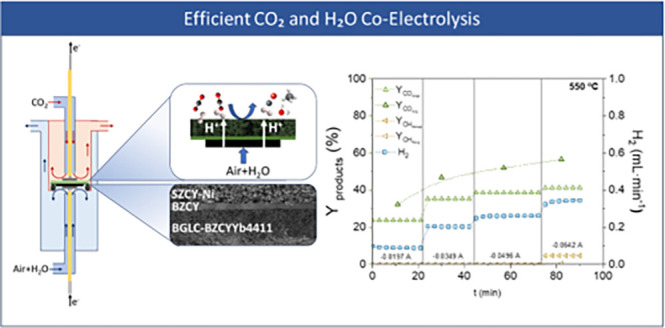

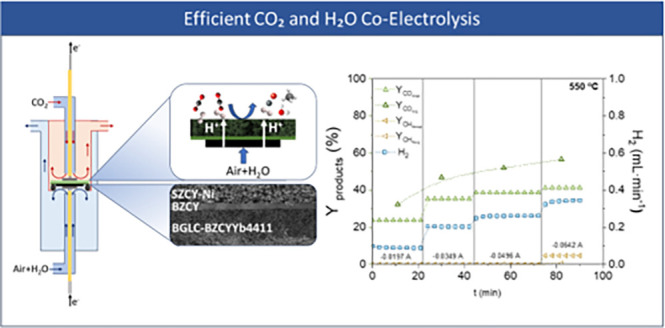

## 1 Introduction

The global energy demand is primarily satisfied by nonrenewable sources. In 2021, fossil fuels (including oil, coal, and natural gas) represented 80.3% of the world’s energy consumption.^1^ The issue is the increasing trend in energy consumption, which rises yearly along with CO_2_ emissions. This phenomenon has led to a significant environmental impact that persists over time. While a greater reliance on electric power could potentially decrease the demand for liquid fuels, hydrocarbons would remain essential, particularly for aviation, marine vessels, and vehicles. Additionally, hydrocarbons supply the chemical compounds necessary for various chemical industry sectors. Therefore, it is crucial to develop environmentally and economically sustainable methods for producing organic molecules.

Electrochemical devices powered by renewable energy offer a promising opportunity to convert CO_2_ into sustainable fuels and chemical feedstock. This process enables the transformation of CO_2_ into valuable chemicals and fuels, thereby contributing to the reduction of greenhouse gas emissions. This approach also facilitates energy storage, which can significantly benefit the energy sector and environment. To date, most research on the co-electrolysis of CO_2_ and H_2_O has focused on solid-oxide electrolysis cells (SOEC),^2–11^ which are operated at high temperatures (600–850 °C). Proton ceramic electrolytic cells (PCECs) are a good alternative due to their high ionic conductivity at intermediate temperatures (400–650 °C).^12–18^ The benefits of proton ceramic electrochemical reactors (PCERs) for CO_2_ conversion include lower operating temperatures, improved electrochemical efficiency through thermal coupling of endothermic and exothermic reactions on both electrolyte sides, and the direct production of dry, high-value chemicals, simplifying downstream processing.^13,19,20^

The most studied proton-conducting ceramic material to date is Y-doped Barium Zirconate Cerate (BZCY), as it combines the high proton conductivity of BCY^21^ with the high stability of BZY.^22,23^ To increase the stability of BZCY in CO_2_ atmospheres, studies have shown that the composition must have a high Zr/Ce ratio, but this results in a loss of conductivity.^24–26^ Choi et al.^27^ introduced a new stoichiometry, BaZr_0.4_Ce_0.4_Y_0.1_Yb_0.1_O_3‑δ_ (BZCYYb4411) and reported that substituting part of the Y with Yb significantly increased conductivity and improved stability, particularly in terms of CO_2_ tolerance. Although BZCY-based ceramic proton conductors can achieve good electrolytic conductivity at intermediate temperatures, the sluggish kinetics of the steam electrode is a major challenge at these temperatures. The double perovskite oxide BaGd_0.8_La_0.2_Co_2_O_6‑δ_ (BGLC) has been investigated as an oxygen electrode for ceramic proton electrolytic cells, as it has a mixed electron and proton conductivity.^28–31^ This is because BaLaCo_2_O_6‑δ_ and BaGdCo_2_O_6‑δ_ perovskites are protonated at intermediate temperatures, as they have been shown to exhibit significant hydration at 300–400 °C.^30^

In our recent work, we demonstrated the potential of process intensification for CO_2_ catalytic conversion using an electrified Ni/BZCY72 tubular protonic membrane reactor, achieving methane yields exceeding 99%.^20^ Building on these findings, the present study aims to integrate hydrogen production (via steam electrolysis) and CO_2_ hydrogenation into a single-step process. Motivated by this approach, we investigate BaGd_0.8_La_0.2_Co_2_O_6‑δ_ as a promising steam electrode material for co-electrolysis in a BZCY-based proton ceramic electrochemical reactor. First, electrochemical impedance spectroscopy (EIS) studies were performed with symmetrical cells and electrodes composed of BaGd_0.8_La_0.2_Co_2_O_6‑δ_ with different protonic materials, BaZr_0.5_Ce_0.3_Y_0.2_O_3‑δ_ and BaZr_0.4_Ce_0.4_Y_0.1_Yb_0.1_O_3‑δ_. After selecting the steam electrode material, the entire co-electrolysis process was assessed in a PCER, combining the electrochemical and catalytic performance to convert CO_2_ and H_2_O.

## 2 Experimental Section

The electrodes were fabricated with commercial powders by mixing BaGd_0.8_La_0.2_Co_2_O_6‑δ_ (BGLC) (Marion technologies) with two different proton conductors BaZr_0.5_Ce_0.3_Y_0.2_O_3‑δ_ (BZCY532) (CerPoTech) and BaZr_0.4_Ce_0.4_Y_0.1_Yb_0.1_O_3‑δ_ (BZCYYb4411) (CerPoTech). Symmetrical cells were then produced by screen printing, coating the electrodes on both sides of the electrolyte, resulting in an active area of 0.64 cm^2^. The inks were prepared using a three-roll mill comprising 50% v/v of each phase, along with terpineol (90%, Sigma-Aldrich) and ethyl cellulose (Sigma-Aldrich). The electrodes were sintered at 950 °C for 7 h in synthetic air. To ensure effective current collection, Ag paste was brush-painted in a mesh pattern on the sintered electrodes and subsequently calcined at 700 °C for 2 h. In summary, the following two cell configurations were produced: Ag/BGLC-BZCY532/BZCY532/BGLC-BZCY532/Ag and Ag/BGLC-BZCYYb4411/BZCYYb4411/BGLC-BZCYYb4411/Ag.

XRD measurements were conducted using CubiX FAST equipment employing CuKα_1,2_ radiation and an X’Celerator detector configured in Bragg–Brentano geometry, covering a 2θ range from 20 to 90°. The XRD patterns were analyzed using the X’Pert Highscore Plus software (PANalytical). The microstructure of the electrodes and the electrode–electrolyte interface was investigated using field-emission scanning electron microscopy (FE-SEM) with a Zeiss Ultra 55 instrument.

Transport properties of the steam electrodes were evaluated by electrochemical impedance spectroscopy (EIS) using the manufactured symmetrical cells. Electrochemical impedance spectroscopy (EIS) was measured in the frequency range of 1 MHz to 0.01 Hz, and an alternating current (AC) signal of 30 mV was recorded using a Solartron 1470E equipped with a 1455A frequency response analyzer (FRA) module. The ZView software was used to analyze and fit the impedance spectra. EIS measurements were performed in a quartz reactor using Pt meshes and evaluated in a temperature range between 600 and 450 °C under wet (3% H_2_O) oxidizing conditions (*p*O_2_ ranging from 1 to 0.2 bar diluted in Ar) with a total flow of 100 mL·min^–1^.

Electrode-supported cells were developed using a sequential tape-casting process utilizing a KARO cast 300–7 microtape casting device (KMS Automation GmbH, Germany). The manufacturing procedure has been described in previous studies.^18^ In summary, the support electrode slurry was formulated using commercial NiO powder and SrZr_0.5_Ce_0.4_Y_0.1_O (SZCY541) in a 60:40 weight ratio. This blend was supplemented with ethanol and methyl ethyl ketone, along with various compounds that served as dispersing agents, plasticizers, and binders. The electrolyte slurry, composed of BaZr_0.44_Ce_0.36_Y_0.2_O_3‑δ_ (BZCY4436), was prepared following an established method,^32,33^ and cast onto a silicone-coated polymeric foil. After drying at ambient temperature, the NiO-SZCY541 slurry was applied over the electrolyte layer and dried for 6 h. Subsequently, the green tapes were cut to the specified dimensions and sintered at 1350 °C for 5 h, resulting in the formation of the half-cell BaZr_0.44_Ce_0.36_Y_0.2_O_3‑δ_/NiO-SZCY541. To complete the cell assembly, BGLC-BZCYYb4411 electrode ink was applied via screen printing and subjected to calcination at 950 °C for 7 h in synthetic air. Finally, a silver collector was incorporated and calcined at 700 °C for 2 h. The co-electrolysis of CO_2_ and H_2_O in the manufactured PCER (BGLC-BZCYYb4411/BaZr_0.44_Ce_0.36_Y_0.2_O_3‑δ_/NiO-SZCY541) was performed in a double-chamber quartz reactor. The cell was then sealed using an Ag-based gasket. An alumina multibore with current collectors of Pt and a thermocouple was placed inside both chambers, which is also used as the gas inlet (Section 3.2). Before the reaction test, the cathode (Ni-SZCY541 electrode) was reduced at 800 °C in an atmosphere of 10 mL·min^–1^ of H_2_ and 90 mL·min^–1^ of Ar. First, the full PCER was electrochemically characterized in the steam electrolysis mode through EIS, *i–V* analysis, and Faradaic efficiency evaluation. Subsequently, the integrated co-electrolysis process was studied by combining hydrogen production via steam electrolysis and CO_2_ hydrogenation. 10 mL·min^–1^ of Ar and 10 mL·min^–1^ of a gas mixture composed of 1% CO_2_ and 5% He in Ar were fed into the reaction chamber (Ni-SZCY541 electrode) while 50 mL·min^–1^ of humidified air (3% H_2_O) was fed into the steam chamber. The performance of the cell under co-electrolysis conditions was evaluated over a temperature range of 550–650 °C. The gas composition at the reaction chamber outlet was analyzed using a Micro-GC 990 (Agilent) gas chromatograph equipped with Molsieve 5A and PoraPlot-Q glass capillary modules.

## 3 Results and Discussion

### 3.1 Steam Electrodes Electrochemical Characterization

The electrochemical performance of the composite electrodes BGLC-BZCYYb4411 and BGLC-BZCY532 was evaluated in symmetrical cells using electrochemical impedance spectroscopy (EIS). This analysis was performed under oxidizing conditions in wet air atmosphere saturated with 3% H_2_O over a temperature range of 600 to 450 °C. The impedance spectra were fitted with the equivalent circuit L­(R_0_)­(R_1_Q_1_)­(R_2_Q_2_). The polarization resistance was calculated by adding the contributions from the two types of resistances (R_P_ = R_1_ + R_2_), where R_1_ corresponds to the resistance contribution associated with high frequencies (HF), and R_2_ corresponds to the resistance contribution associated with low (LF) frequencies. Figure 1a shows the Arrhenius plot of the total polarization resistance (R_p_) as a function of temperature for both composite electrodes, BGLC-BZCYYb4411 and BGLC-BZCY532. Both electrodes show similar performance in air, although BGLC-BZCY532 presents a slightly lower activation energy, resulting in lower polarization resistance values at lower temperatures. It should be noted that the impedance spectra were not corrected for a parallel electronic conduction path in the electrolyte. Consequently, the spectra were not deconvoluted into separate ionic and electronic contributions, i.e. protons, oxide ions and electron holes using such a correction.^34^ This may lead to an underestimation of the apparent polarization resistances, mainly at high temperatures, and, consequently, to an overestimation of the activation energies.^31^

**Figure 1:**
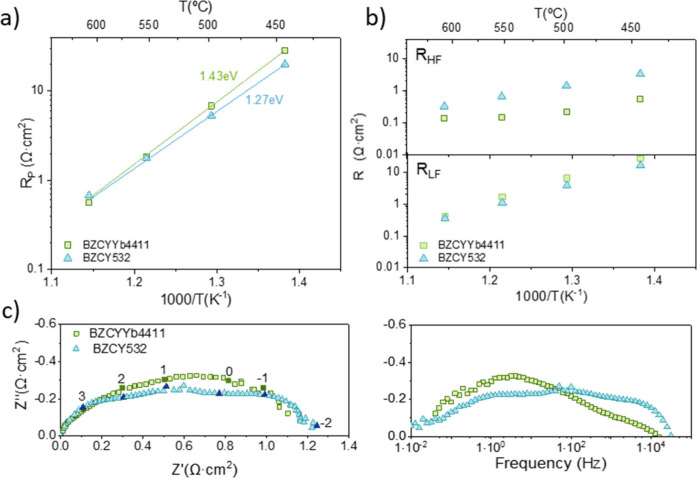
(a) Polarization resistance (R_p_) and (b) resistance contributions at HF (RHF) and LF (RLF) of symmetrical cells as a function of temperature under wet synthetic air. (c) Nyquist and Bode plots of the symmetrical cells at 500 °C under wet air. The numbers represent the logarithm of the frequency of each marked point.

Figure 1b presents the resistance contribution at high frequencies (hereafter referred to as R_HF_) and low frequencies (hereafter referred to as R_LF_). The HF contribution is typically assigned to the charge transfer reactions.^35,36^ Conversely, LF contributions are generally associated with elemental reactions at the electrode surface, particularly the adsorption/desorption processes related to surface exchange kinetics.^37^ Both electrodes exhibited a higher resistance contribution at low-medium frequencies (R_LF_), suggesting that surface processes are the limiting step in electrode performance.^35^

Figure 1c shows the Nyquist and Bode plots obtained at 500 °C in wet air for symmetrical cells with BGLC-BZCYYb4411 and BGLC-BZCY532 composite electrodes. In these graphs, the two contributions at high and low frequencies are clearly observed. At low-medium frequencies (R_LF_), contributions occur with frequencies of 10^–1^-100 Hz and capacitance of 10^–1^-10^–3^ F/cm^2^, while high frequencies (R_HF_) contributions appear with frequencies above 1–10 kHz and capacitances between 10^–4^-10^–6^ F/cm^2^.^25^ A comparison of the Nyquist and Bode plots shows that the BGLC-BZCYYb4411 electrode presents a significantly higher contribution at low frequencies under humid air conditions. These trends remain consistent across all measured temperatures, as shown in Figures S1–S4. The lower magnitude of the HF contribution observed for BZCYYb4411 may be related to a better interface between BGLC-BZCYYb4411 and the BZCYYb4411 electrolyte than that between the BGLC-BZCY532 composite and the BZCY532 electrolyte. It should be noted that electrode performance depends on several parameters, such as its electrochemical properties and the electrode–electrolyte interface, among others. In the present study, two different electrolytes were employed with different stoichiometries and, consequently, different partial conductivities. This may influence the electrode performance; however, this effect has not been specifically investigated in this work.

Considering the reduced low-frequency resistance and enhanced electrode–electrolyte interface, the BZCYYb4411 composite electrode was selected for further studies and its integration into a full co-electrolysis cell. Additional electrochemical characterization of the BGLC-BZCYYb4411 electrode as a function of *p*O_2_ (at a constant *p*H_2_O of 0.03 atm) was conducted to study the nature of the different resistive contributions.

As previously reported for different electrodes used in proton-conducting cells,^38–40^ the electrode resistance is proportional to *p*O_2_ and *p*H_2_O, as indicated in eq 1 being the exponents *m* and *n* directly related to the elementary steps occurring at the electrode.^39,41^

1 $$R_{i}\propto pO_{2}^{-m_{i}}pH_{2}O^{-n_{i}}$$

Several studies have reported the different reaction steps that take place in a steam electrode in both fuel cell (PCFC) and electrolysis (PCEC) mode.^40,42–45^ Typically, the sequence of steam electrode reactions in PCEC and PCFC is reversed. In the PCEC mode, when current is applied, steam is split into oxygen and protons at the steam electrode, while hydrogen is produced at the hydrogen electrode. Table 1 presents the elementary reaction steps in PCEC steam electrodes as reported by Kobayashi et al.^40^

**Table 1:** Elementary Steam Electrode Reaction Steps and Their Reaction Order with Respect to Oxygen Partial Pressure (*m*) and Steam Partial Pressure (*n*) for PCECs

Elementary reaction		*m*	*n*
Step 1	H_2_O(g) → H_2_O(TPB)	0	1
Step 2	H_2_O(TPB) → OH^–^(TPB) + H^+^(TPB)	0	1
Step 3	OH^–^(TPB) → O^2–^(TPB) + H^+^(TPB)	0	1/2
Step 4	H^+^(TPB) → H^+^(elec)	0	1/2
Step 5	O^2–^(TPB) + h^+^ → O^–^(TPB)	0	0
Step 6	O^–^(TPB) → O^–^(ad)	1/4	0
Step 7	O^–^(ad) + h^+^ → O(ad)	3/8	0
Step 8	2O(ad) → O_2_(g)	1	0

Figure 2 a shows the Nyquist and Bode plots as a function of *p*O_2_, ranging from 1 to 0.2 bar at 450 °C. Figure 2 b plots the isothermal analysis at 450 °C for R_p_, R_HF_, and R_LF_ as a function of *p*O_2_, whereas the capacitances associated with each contribution are shown in Figure 2c.

**Figure 2:**
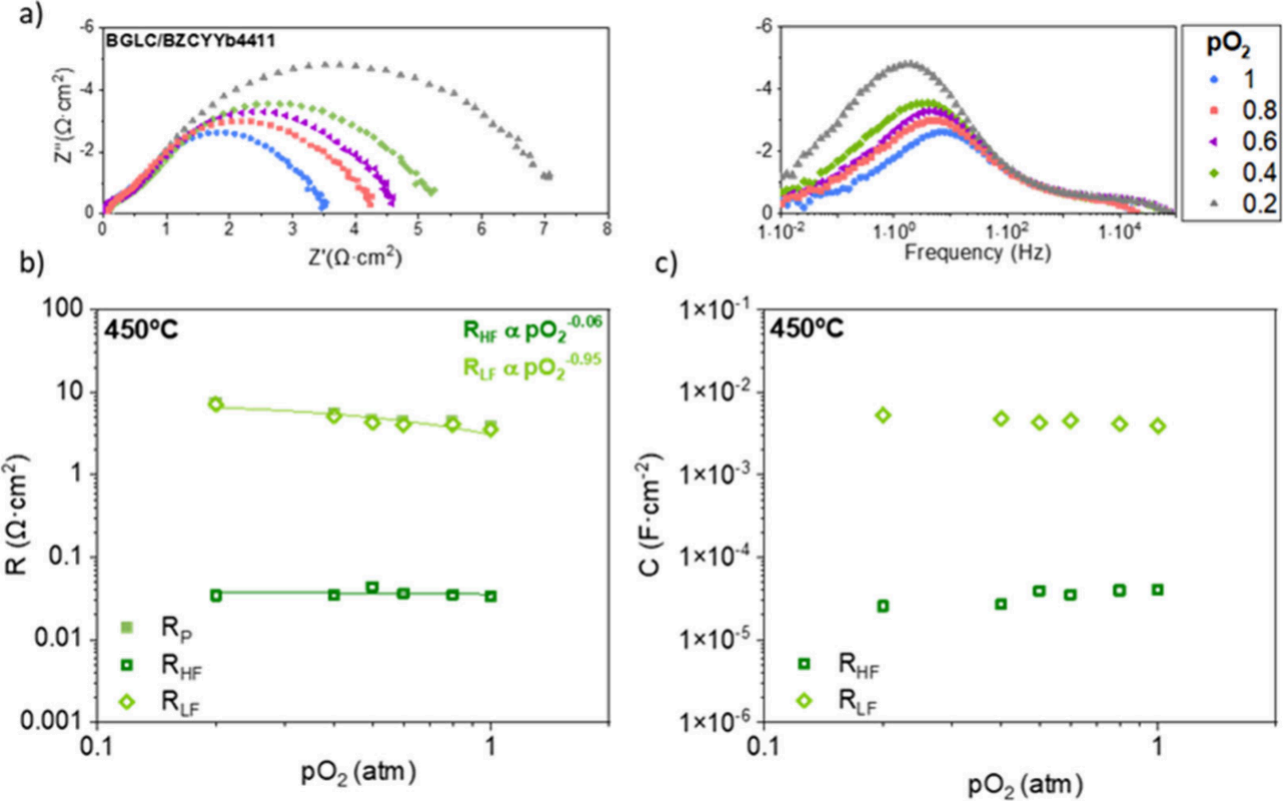
(a) Nyquist and Bode plots, (b) polarization resistance, and (c) the corresponding capacitances for the BGLC-BZCYYb4411 electrode measured at 450 °C under different *p*O_2_.

A decrease in the low-frequency resistance contribution is observed when the partial pressure of oxygen increases (*p*O_2_^–0.95^), whereas R_HF_ remains nearly constant (*p*O_2_^–0.06^).

Then, the HF contribution presents an *m* value of 0.06, whereas the corresponding value for the LF contribution is 0.95. Based on this dependency, the HF contribution can be related with the dissociative adsorption of H_2_O (water splitting reaction) at the triple phase boundary (TPB) and the associated charge transfer reactions (Step 1–5).^26,27,40^ This assignment is in agreement with the obtained associated capacitance of 10^–5^ F·cm^–2^.^46^ On the other hand, the LF contribution can be related to the O_2_ desorption (step 8) taking into account both the *p*O_2_ dependence and its corresponding capacitance of around 10^–3^ F·cm^–2^.^24^

Figure 3 displays the XRD patterns of the composite electrodes after testing, as well as those of the individual phases as reference. The XRD patterns show that both composite electrodes, BGLC-BZCYYb4411 (Figure 3a) and BGLC-BZCY532 (Figure 3b), maintained their integrity under the operating conditions used. No secondary phase formation was detected in the XRD patterns; only the BGLC phase, the corresponding proton-conducting phase (BZCYYb4411 or BZCY532), and Ag used as current collector (indicated by asterisks) can be identified. SEM cross-sectional images of the BGLC-BZCYYb4411 and BGLC-BZCY532 electrodes after the electrochemical testing are shown in Figures 3a and 3b, respectively. Both electrodes exhibit similar morphology with a particle size of 0.3–0.5 μm, porosity, and electrode thickness of 14–16 μm.

**Figure 3:**
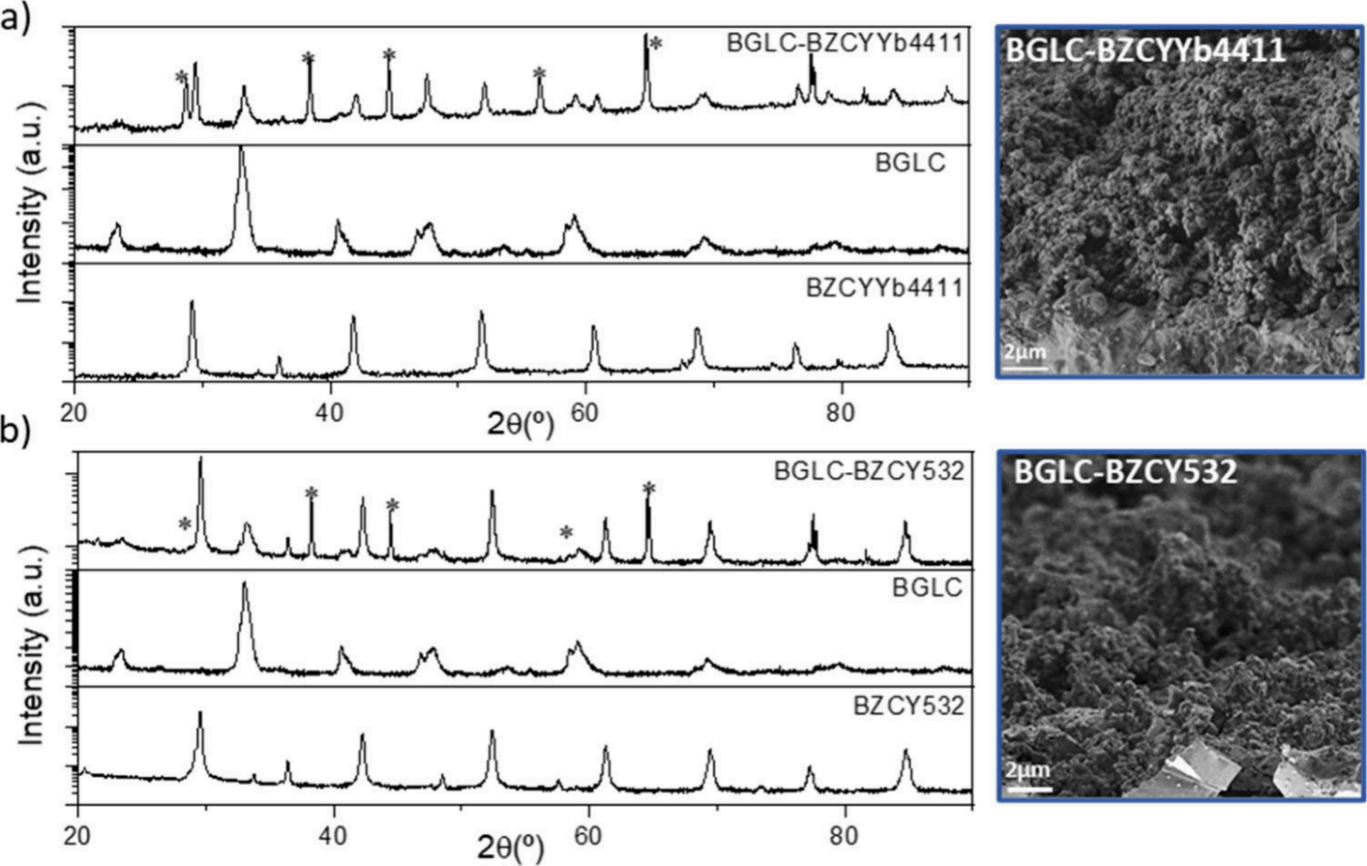
XRD patterns and SEM cross-sectional images of the electrodes BGLC-BZCYYb4411 (a) and BGLC-BZCY532 (b) after the electrochemical measurements. Peaks assigned to Ag (used as the current collector) are indicated by asterisks.

### 3.2 CO_2_ and H_2_O Co-Electrolysis in a PCER

Co-electrolysis of CO_2_ and H_2_O was conducted from 650 to 550 °C at 1 bar with a PCER composed of a BGLC-BZCYYb4411 steam electrode deposited on a BaZr_0.44_Ce_0.36_Y_0.2_O_3−δ_/Ni-SrZr_0.5_Ce_0.4_Y_0.1_O_2.95_ half-cell. The steam chamber, where electrolysis occurs, was fed with wet air (3% H_2_O), while dry CO_2_ was introduced into the reaction chamber. The protons produced by steam electrolysis are injected into the reaction chamber through the electrolyte, where H_2_ evolves and reacts with CO_2_ on the Ni electrode surface, i.e., acting as a catalyst for the CO_2_ reduction reaction (Figure 4). The reverse water gas shift (RWGS) reaction is favored under the selected operating conditions, i.e., high temperature and atmospheric pressure, producing predominantly syngas formation.

**Figure 4:**
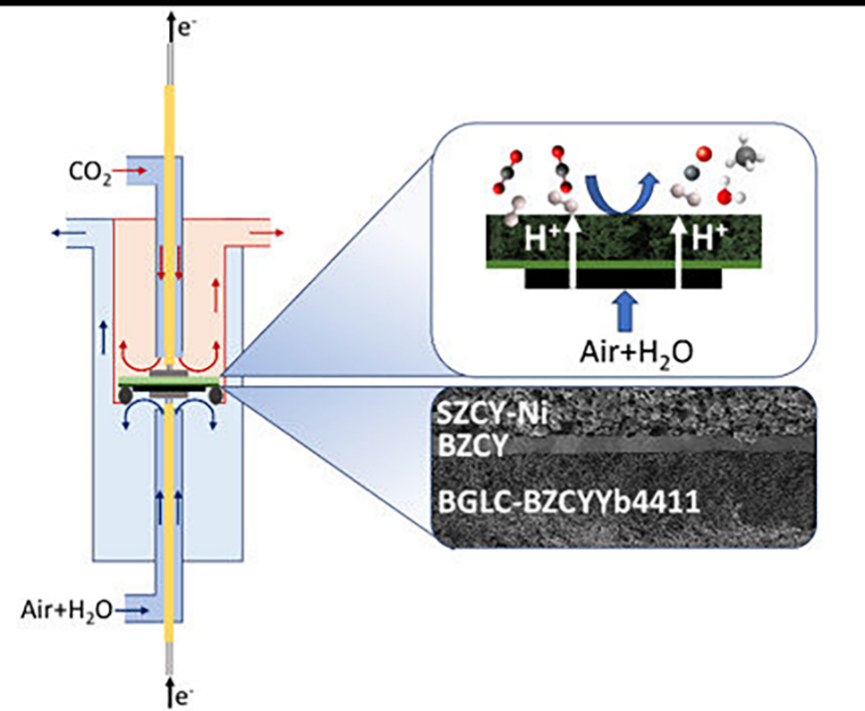
Reactor configuration for the CO_2_ and H_2_O co-electrolysis with a scheme of the working principle and SEM image of the PCER assembly.

First, the electrochemical properties of the cell were characterized for H_2_O electrolysis over a temperature range from 650 to 450 °C by feeding a stream of 100 mL·min^–1^ of humidified air (3% H_2_O) into the steam chamber, and 10 mL·min^–1^ of H_2_ and 90 mL·min^–1^ of Ar into the reaction chamber. Figure 5a shows the *I*–*V* curves at various temperatures, ranging from 650 to 450 °C. At all temperatures, the open circuit voltage (OCV) is approximately 1 V, close to the theoretical value for the given operating conditions. Higher temperatures allow operation at higher current densities; applying up to a current density of 300 mA·cm^–2^ at 650 °C without exceeding 1.8 V. Lower operating temperatures reduce the applicable current density due to the higher area-specific resistance (ASR). This trend is illustrated in Figure 5b, where the total area-specific resistance (ASR) is shown as a function of the temperature obtained from EIS measurements at OCV (Figure S5). The ASR was calculated as the sum of the ohmic and the polarization resistances. At 650 °C, the total ASR was 2.9 Ω·cm^2^, increasing to 21.0 Ω·cm^2^ at 450 °C.

**Figure 5:**
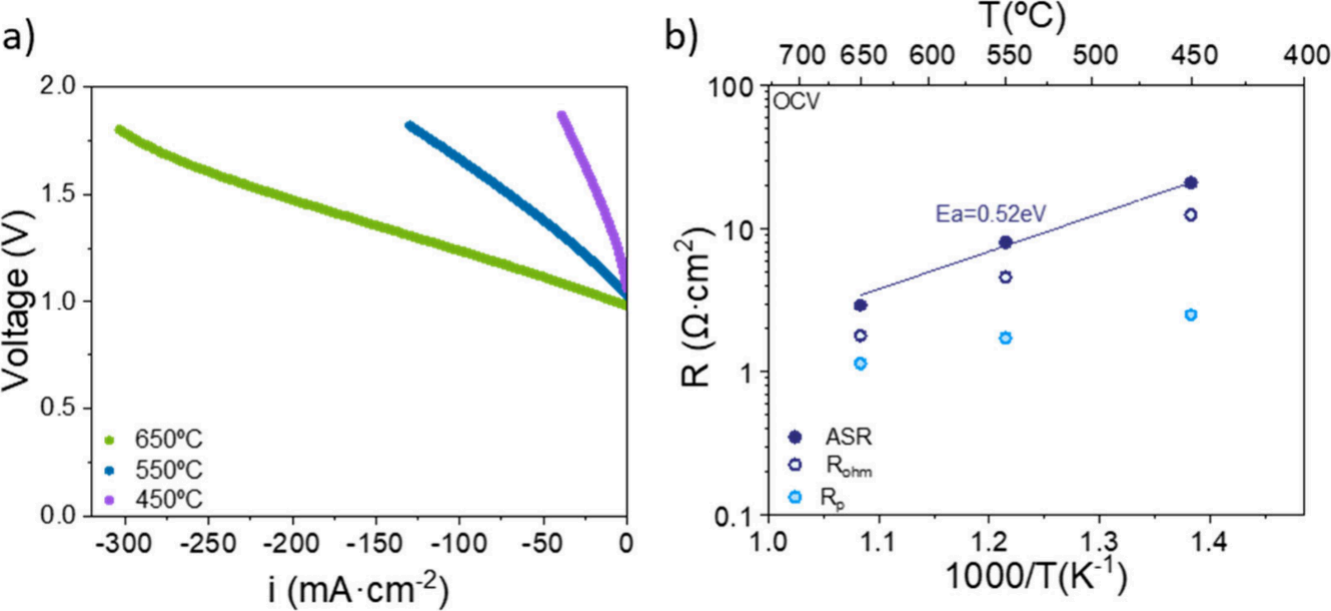
(a) Current–voltage curves, (b) total resistance (ASR), R_p_, and R_ohm_ obtained from EIS for steam electrolysis in a PCER as a function of the temperature from 650 to 450 °C.

Steam electrolysis experiments were conducted at 650 °C (Figure 6a), 550 °C (Figure 6b), and 450 °C (Figure S6). At 650 °C, a maximum current density of 250 mA·cm^–2^ (0.159 A) was applied, obtaining a H_2_ flow of 0.75 mL·min^–1^. The Faradaic efficiency ranged from 74% to 64%, decreasing as the applied current density increased. At 550 °C, due to the higher ASR of the cell, the maximum applied current density during electrolysis was limited to 100 mA·cm^–2^ (0.064 A). Despite this, the Faradaic efficiency remained constant at 100% for the two tested current densities, giving rise to significant H_2_ production. Although BZCY4411 does not exhibit high *p*-type electronic conductivity under these conditions, the contribution of the electronic transport decreases with lower operating temperatures, resulting in higher Faradaic efficiencies.^47^

**Figure 6:**
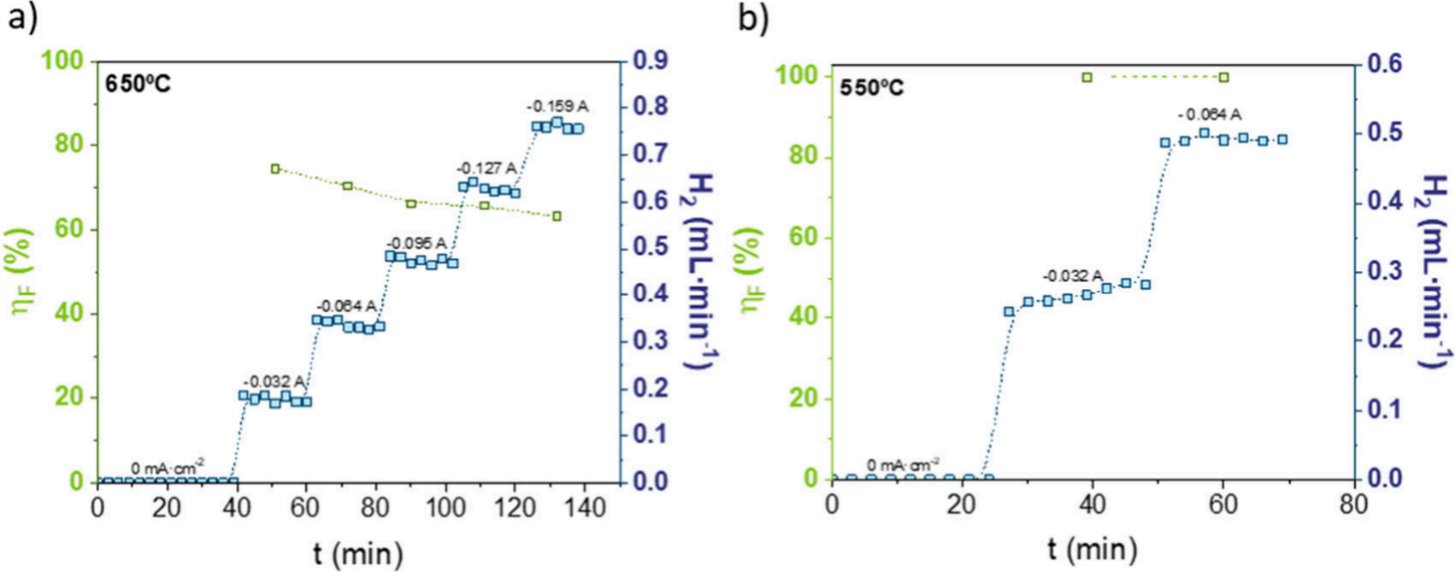
H_2_ flow (blue dots) as a function of the applied current and the corresponding Faradaic efficiency (green dots) in the PCER at 600 °C (a) and 550 °C (b). Faradaic efficiency is the average value of all the data obtained.

The co-electrolysis reaction of CO_2_ and H_2_O was also investigated at 650 and 550 °C. The catalytic performance of the PCER was evaluated based on the H_2_ produced, and the CO and CH_4_ yield as a function of the applied current and time (Figure 7). The CO_2_ reduction reaction depends on the temperature, pressure, and H_2_/CO_2_ ratio.^13^ Under the operating conditions studied, high temperature and low pressure, the RWGS reaction is expected to be favored, as shown in Figure S7, where the CO_2_ conversion and CO and CH_4_ selectivity predicted by the thermodynamic equilibrium are plotted as a function of the H_2_/CO_2_. In fact, only CO was produced for the different tested conditions, except at the highest H_2_/CO_2_ ratio at 550 °C, where a methane yield of 4.7% is obtained. On the other hand, CO yield increases with the H_2_/CO_2_ ratio that is modulated with the applied current, i.e., higher ratios are obtained by increasing the applied current. Therefore, under the tested conditions, syngas formation is the primary reaction, in agreement with the thermodynamic calculations included in Figure 7 for comparison.

**Figure 7:**
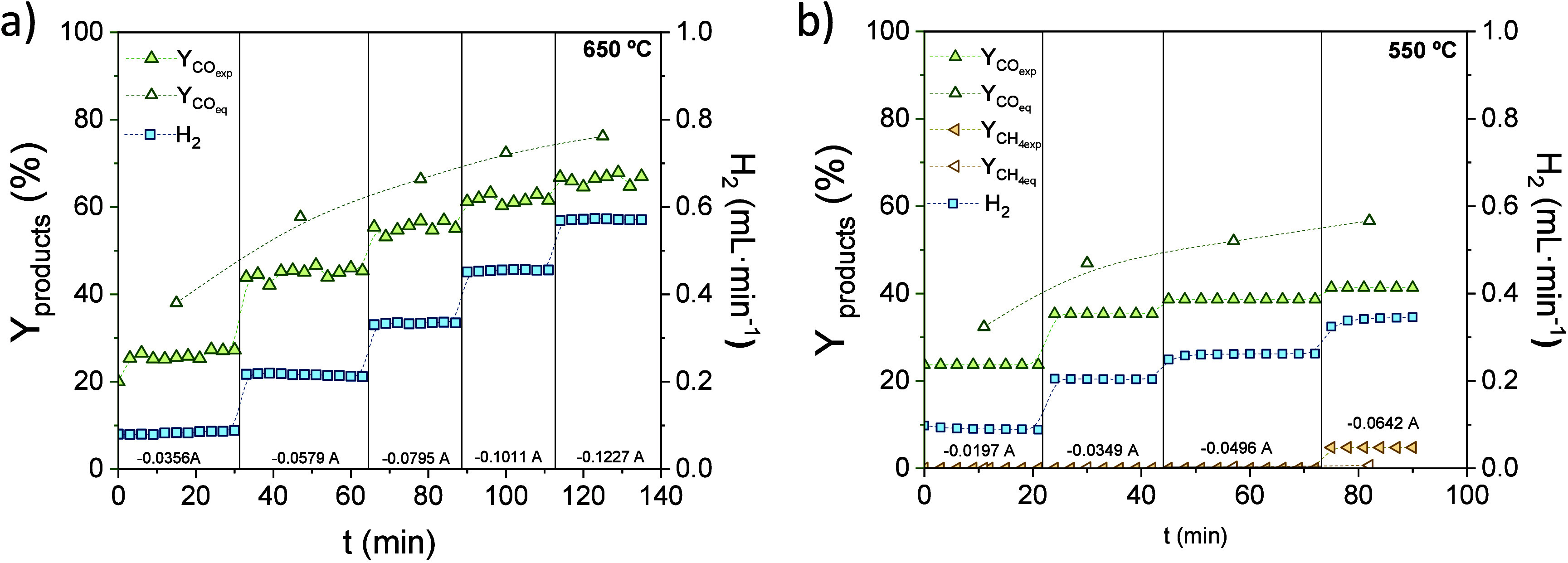
Co-electrolysis performance: CO and CH_4_ yield, and H_2_ production as a function of the applied current in a PCER at (a) 650 °C and (b) 550 °C. Thermodynamic values (empty symbols) have been added for comparison.

Moreover, the H_2_/CO_2_ conversion ratio can be tuned by adjusting the applied current density, demonstrating the operational flexibility of the PCER technology.

The integrity of the PCER after testing was assessed using SEM and XRD. The cross-sectional SEM image (Figure 8a) at the steam electrode (BGLC-BZCYYb4411) and the electrolyte (BZCY4436) interface shows a good attachment and porosity of the steam electrode, with uniform particles of ca. 50 nm and no segregation. The electrolyte has a thickness of ∼ 7 μm, and that of the BGLC-BZCYYb4411 electrode is ∼ 30 μm. Figure 8b shows the XRD pattern of the steam electrode after the reaction. The XRD patterns show the characteristic peaks of the steam electrode (BGLC and BZCYYb4411) and the electrolyte (BZCY4436), with no detectable secondary phase formation, proving that the cell remained stable after the reaction. Although no coke formation was observed during co-electrolysis, the CO_2_ electrode was characterized using Raman spectroscopy to examine whether coke formation occurred during the reaction (Figure 8c). A small amount of coke was detected, as indicated by the Raman characteristic peaks at 1346.6 cm^–1^ (D-band peak) and 1589.9 cm^–1^ (G-band peak), probably formed when operating at a low H_2_/CO_2_ ratio.^48,49^

**Figure 8:**
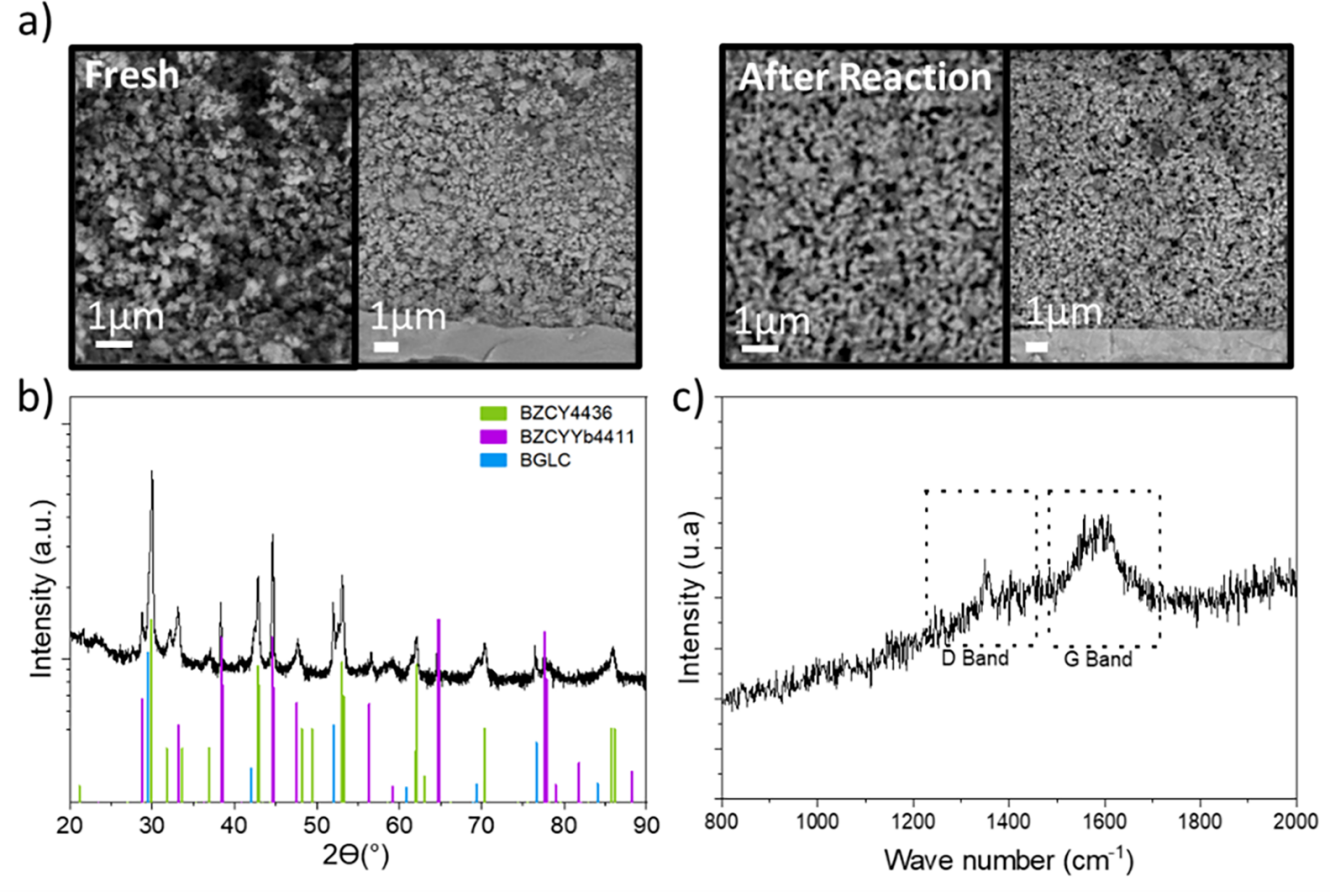
(a) SEM cross-sectional images of the steam electrode–electrolyte interface before and after testing. (b) XRD pattern of the PCER after reaction. (c) Raman spectra of D and G peaks of the coke generated in the fuel electrode.

## 4 Conclusions

The present study investigated the CO_2_ reduction reaction coupled with H_2_O electrolysis in proton ceramic electrochemical cells. The focus was on optimizing the performance of the different steam electrode materials and operational conditions. The electrochemical performance of the composite electrodes BaGd_0.8_La_0.2_Co_2_O_6‑δ_ (BGLC) combined with BaZr_0.4_Ce_0.4_Y_0.1_Yb_0.1_O_3‑δ_ (BZCYYb4411) and BaZr_0.5_Ce_0.3_Y_0.2_O_3‑δ_ (BZCY532) was assessed under humidified oxidizing conditions. Both steam electrodes showed very similar performance, with a polarization resistance of approximately 0.6 Ω cm^2^ at 600 °C in humidified air, with BGLC-BZCYYb4411 showing a low-frequency resistance contribution attributed to an improved electrode–electrolyte interface. Both electrodes remained chemically and structurally stable, as confirmed by XRD and SEM analyses, with no formation of secondary phases or observable microstructural degradation. Additional electrochemical characterization of the BGLC-BZCYYb4411 electrode as a function of the *p*O_2_ was performed to better understand the processes limiting its performance, which are associated with the associative oxygen desorption linked to surface exchange kinetics.

The BGLC-BZCYYb4411 electrode was selected to evaluate CO_2_ and H_2_O co-electrolysis in a full electrochemical reactor (PCER). A cathode-supported electrochemical cell consisting of BaGd_0.8_La_0.2_Co_2_O_6‑δ_-BaZr_0.4_Ce_0.4_Y_0.1_Yb_0.1_O_3‑δ_/BaZr_0.44_Ce_0.36_Y_0.2_O_3−δ_/Ni-SrZr_0.5_Ce_0.4_Y_0.1_O_2.95_ was employed, and its performance under electrolysis and co-electrolysis was assessed. Steam electrolysis was evaluated at 650 and 550 °C. The highest H_2_ flow rate of 0.75 mL·min^–1^ was achieved at 650 °C; however, the corresponding Faradaic efficiency was moderate (approximately 64%). When the temperature was reduced to 550 °C, the Faradaic efficiency increased to 100%. Subsequently, co-electrolysis was performed, with CO being the main product, which was ascribed to the operating conditions (temperature and pressure) that favored the RWGS reaction. Post-mortem analysis by SEM and XRD confirmed the stability of the PCER under the tested conditions.

These results evidence the great potential of these reactors for CO_2_ conversion. In fact, by tuning the operating conditions and H_2_/CO_2_ ratio, which is controlled by the applied current density, the CO_2_ reduction reaction can be directed toward syngas production, favored by higher temperatures, lower pressures, and low H_2_/CO_2_ or CH_4_ production, favored by lower temperatures, higher pressures, and high H_2_/CO_2_, revealing the potential high level of flexibility of the proposed membrane reactor system.

## Supplementary Material



## Notes

Nyquist and Bode plots, *I*–*V* curve under electrolysis conditions, and H_2_ production at 450 °C and thermodynamic equilibrium of the CO_2_ reduction reaction (PDF)
