# Supplementary material for: Efficient CO2 and H2O Co-Electrolysis in a BaZr0.44Ce0.36Y0.2O3‑δ-Based Proton Ceramic Electrochemical Reactor with BaGd0.8La0.2Co2O6‑δ Steam Electrodes
Source: ACS Appl Energy Mater. 2026 Jan 15;9(3):1590–8. doi: 10.1021/acsaem.5c03443 (PMC12892237; doi:10.1021/acsaem.5c03443)
Supplement: Supplementary file 1 [file ae5c03443_si_001.pdf]

Supporting Information

Efficient CO_2 and H_2O Co-Electrolysis in a $BaZr_{0.44}Ce_{0.36}Y_{0.2}O_{3-\delta}$ based Proton Ceramic Electrochemical Reactor with $BaGd_{0.8}La_{0.2}Co_2O_{6-\delta}$ steam electrodes

Elena Barrio-Querol[1], Imanol Quina[1], Maria Fabuel[1], Kwati Leonard[2], Hiroshige Matsumoto[2], José Manuel Serra[1*], Laura Almar[1*], Sonia Escolástico[1*]

[1]Instituto de Tecnología Química (Universitat Politècnica de València-Consejo Superior de Investigaciones Científicas), Av. Los Naranjos s/n 46022 València, Spain

[2] Center for Energy Systems Design (CESD), International Institute for Carbon-Neutral Energy Research (I2CNER), Kyushu University, 744 Motooka, Nishi-ku, Fukuoka, 819-0395, Japan

**lauallia@itq.upv.es*
**jmserra@itq.upv.es*
**soesro@itq.upv.es*

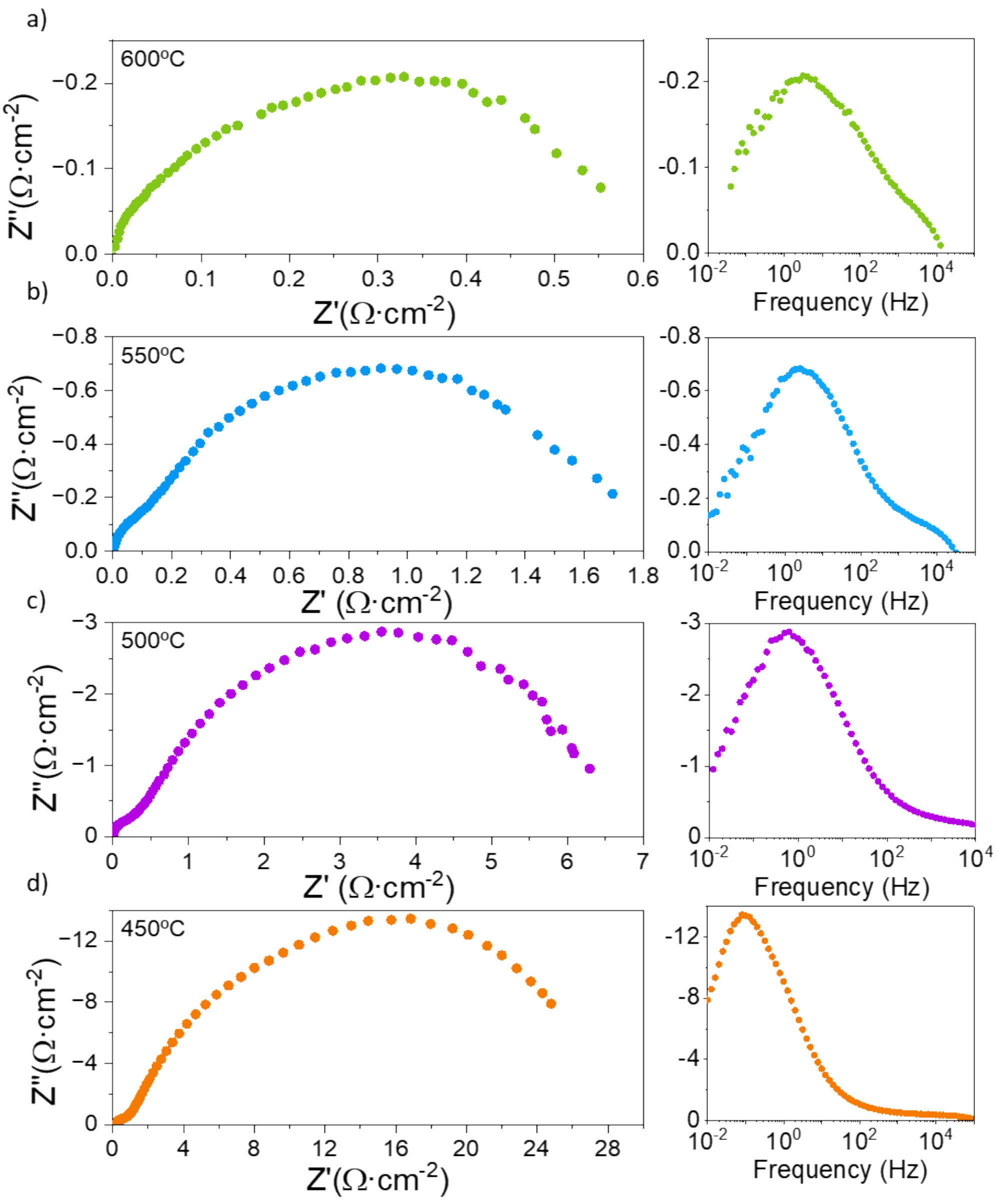

Figure S1. EIS spectra (Nyquist and Bode plot) for the composite electrodes BGLC-BCZYYb4411 measured at 600 °C (a), 550°C (b), 500 °C (c) and 450 °C (d) under wet air.

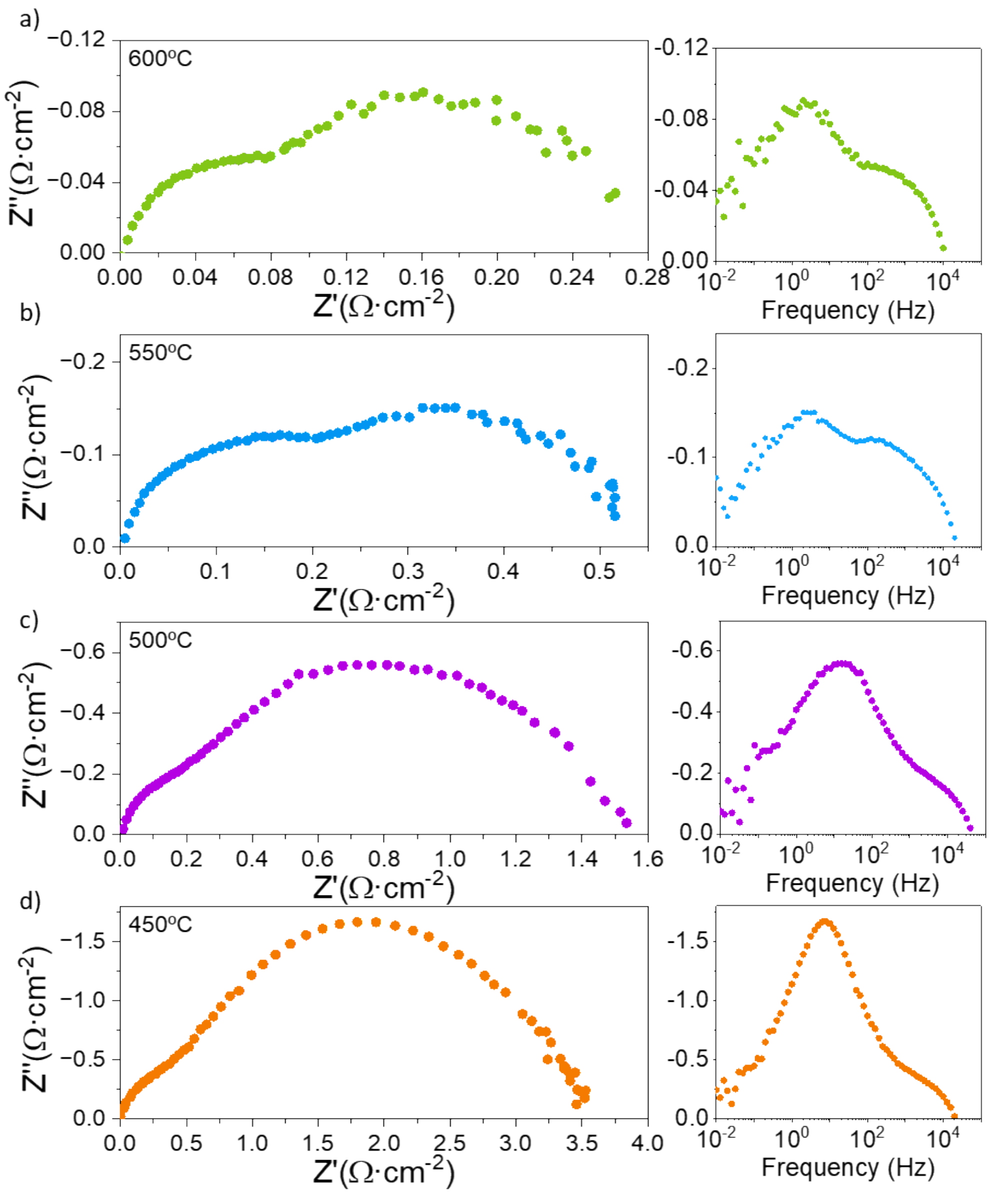

Figure S2. EIS spectra (Nyquist and Bode plot) for the composite electrodes BGLC-BCZYYb4411 measured at 600 °C (a), 550°C (b), 500 °C (c) and 450 °C (d) under wet O_2.

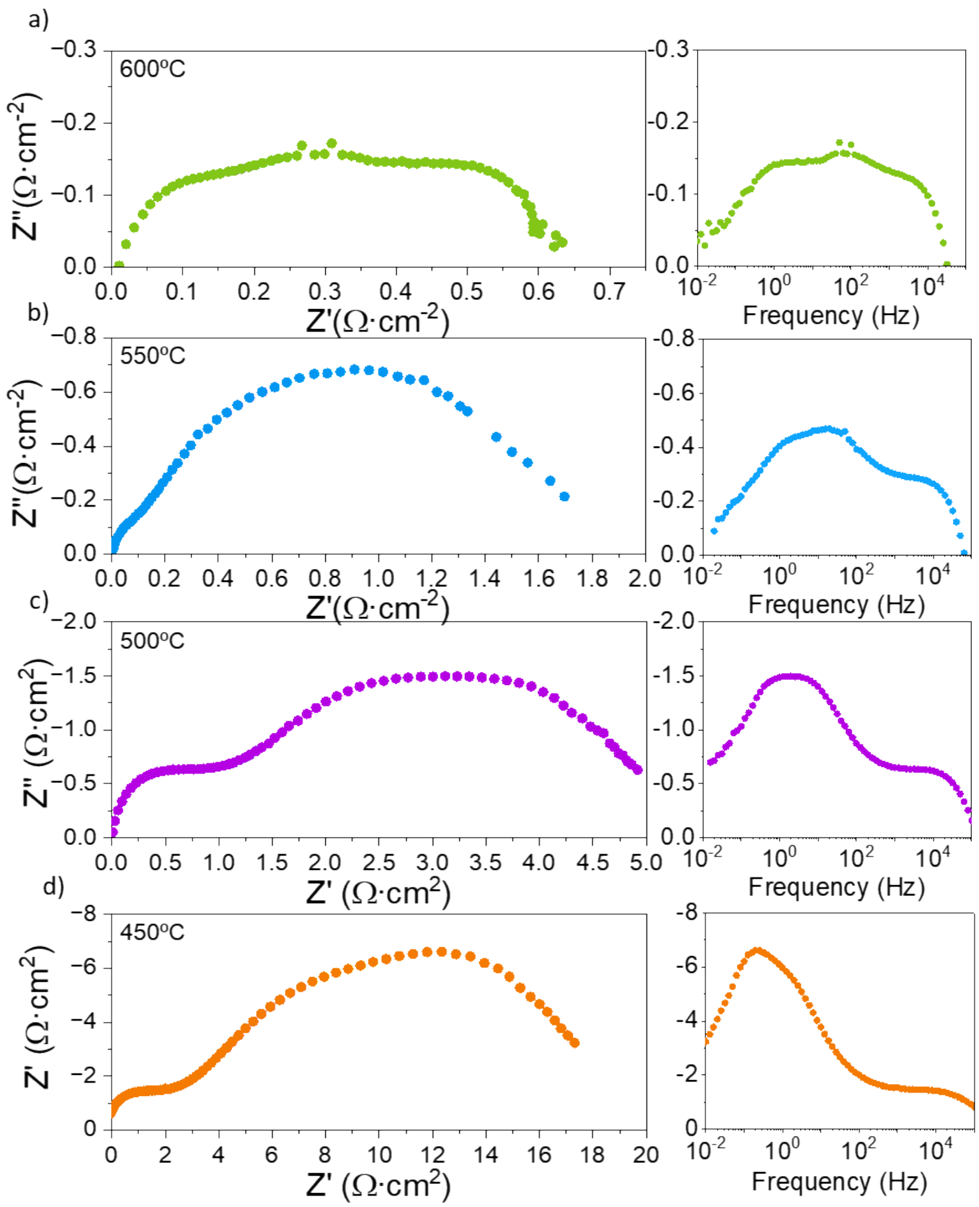

Figure S3. EIS spectra (Nyquist and Bode plot) for the composite electrodes BGLC-BCZY532 measured at 600 °C (a), 550°C (b), 500 °C (c) and 450 °C (d) under wet air.

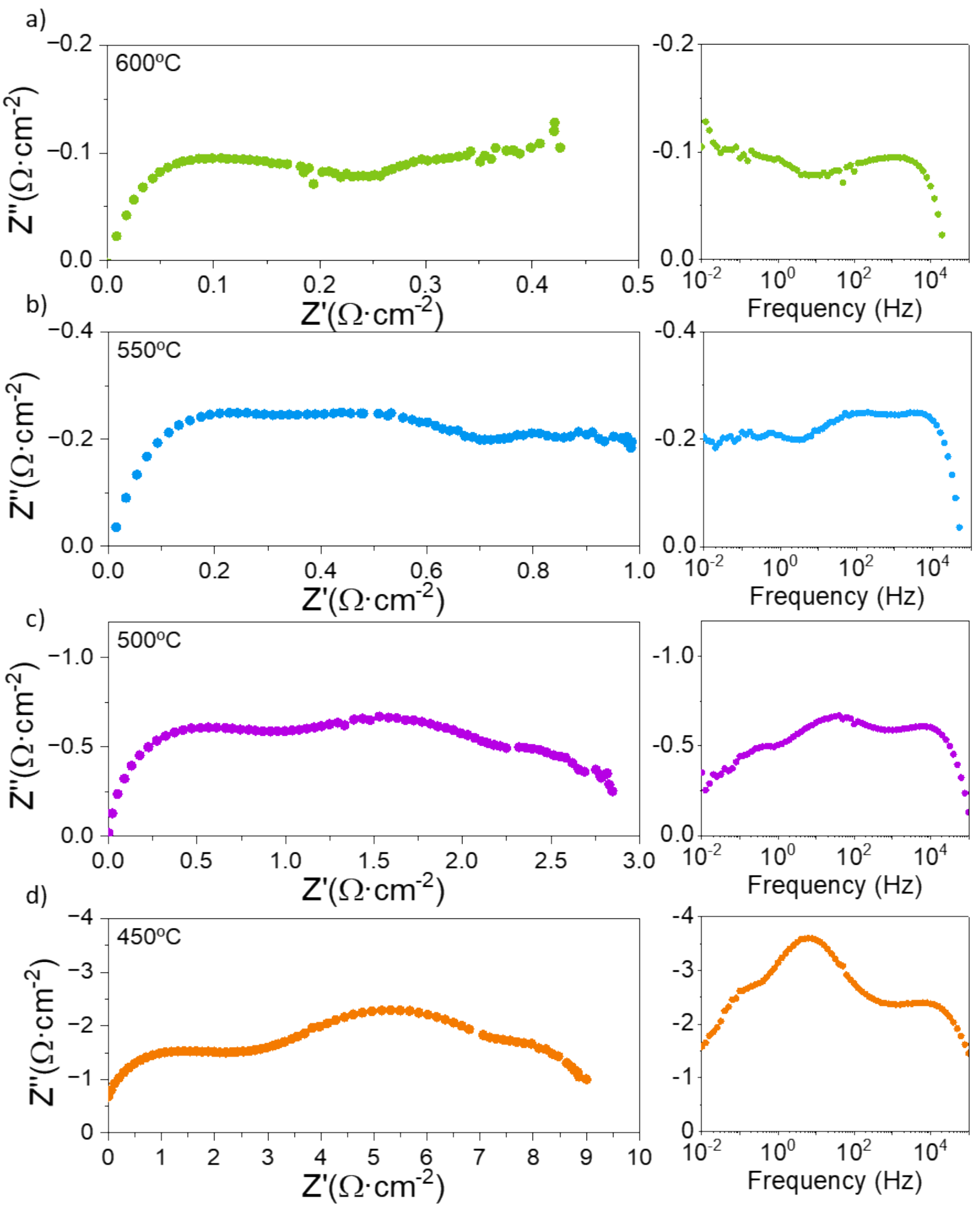

Figure S4. EIS spectra (Nyquist and Bode plot) for the composite electrodes BGLC-BCZY532 measured at 600 °C (a), 550°C (b), 500 °C (c) and 450 °C (d) under wet O_2.

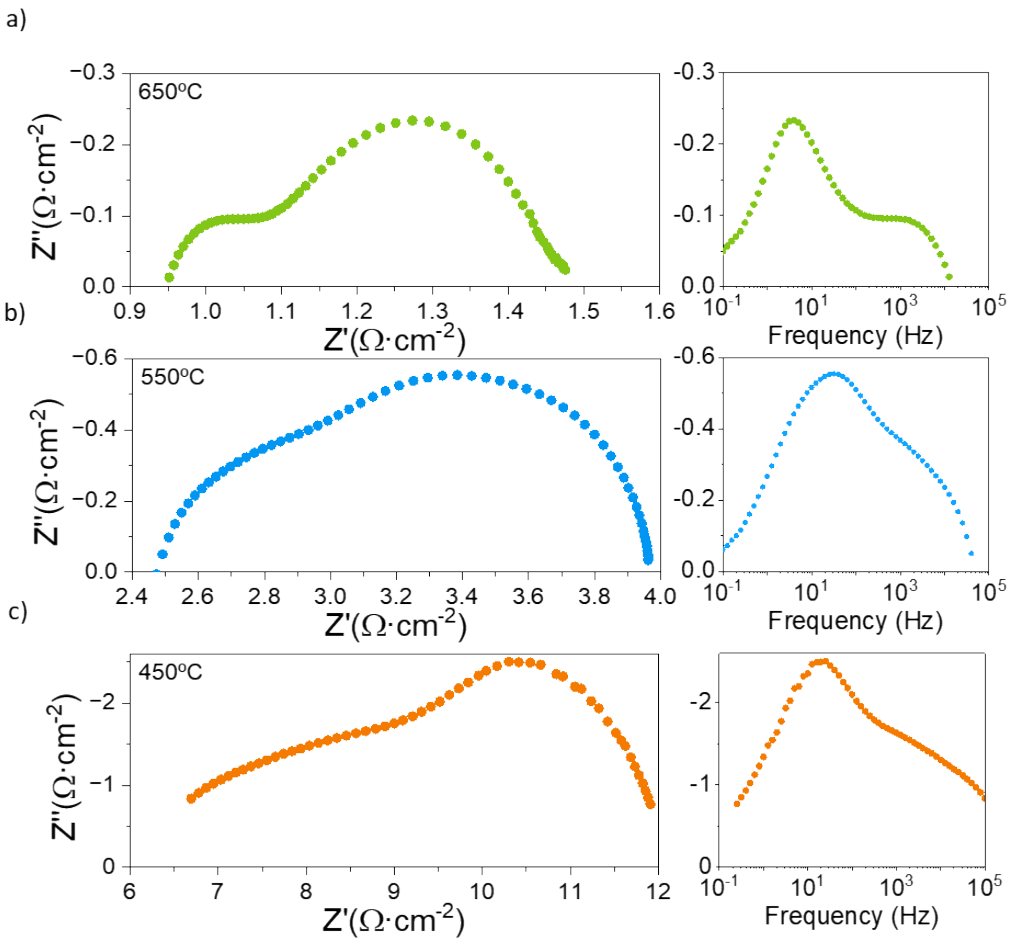

Figure S5. EIS spectra (Nyquist and Bode plot) for the composite electrodes BGLC-BCZYYb4411 measured at 650 °C (a), 550°C (b) and 450 °C (c) under electrolysis condition.

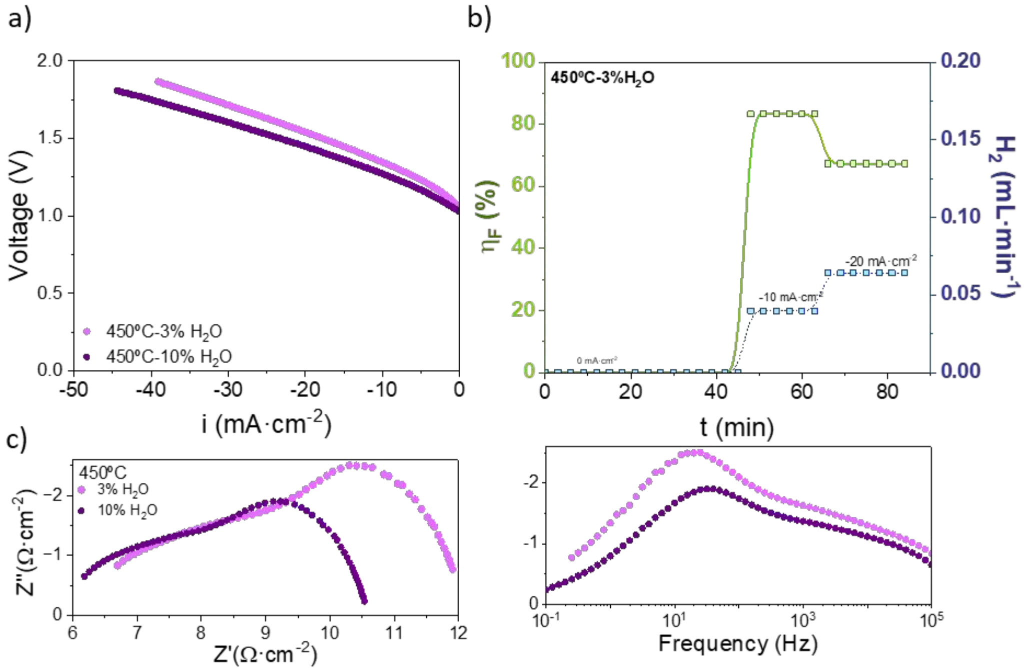

Figure S6. I-V curve (a), H_2 flow as a function of the applied current density and the corresponding Faradaic efficiency (b) and EIS spectra (Nyquist and Bode plot) for the composite electrodes BGLC-BCZY532 measured at 450 °C (c)

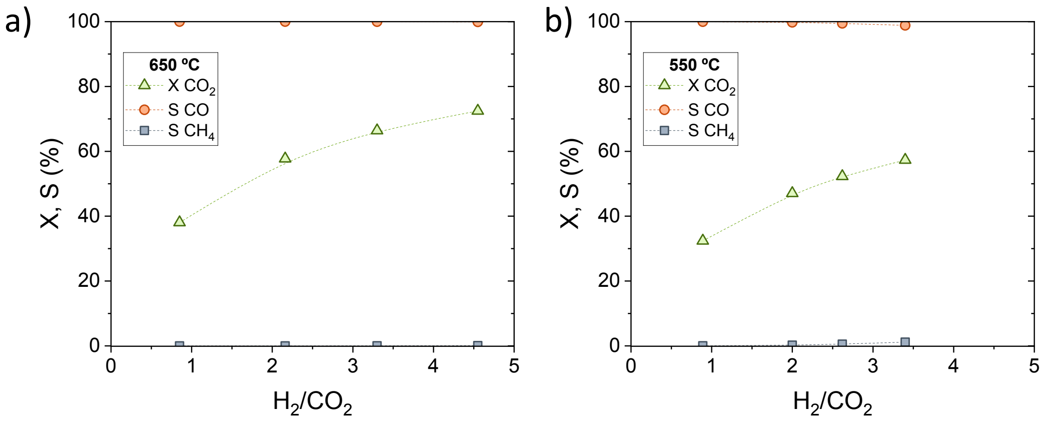

Figure S7. Thermodynamic values for CO_2 conversion and CO and CH_4 selectivity as a function of the H_2/CO_2 ratio at 650 ºC (a) and 550 ºC (b) and 1 bar. Calculations were performed using the operating conditions applied in the experimental tests and the H_2/CO_2 ratios obtained experimentally.
